# Concentration monitoring and dose optimization for infliximab in Crohn’s disease patients: a machine learning-based covariate ensemble model

**DOI:** 10.3389/fimmu.2025.1715241

**Published:** 2025-12-08

**Authors:** Yuewen Chen, Shoutian Zhang, Si Chen, Shaojun Jiang, Shuifang Zhou, Jing Liu, Zhoujie Liu, Rongfang Lin, Jianwen Xu

**Affiliations:** 1 Department of Pharmacy, The First Affiliated Hospital, Fujian Medical University, Fuzhou, China; 2 Department of Pharmacy, National Regional Medical Center, Binhai Campus of the First Affiliated Hospital, Fujian Medical University, Fuzhou, China; 3 Department of Infectious Disease, The First Affiliated Hospital, Fujian Medical University, Fuzhou, China

**Keywords:** Crohn’s disease, infliximab, dose optimization, machine learning, SHAP

## Abstract

**Background:**

Trough concentration of Infliximab (IFX) was associated with its efficacy and toxicity. However, traditional therapeutic drug monitoring often results in suboptimal outcomes because dose adjustments are delayed. We aimed to develop and validate a machine learning (ML) framework to enable real-time trough concentration prediction (pre-infusion point-of-care prediction) and individualized dosing for Crohn’s disease (CD) patients.

**Methods:**

Leveraging data from a retrospective cohort of 274 Chinese CD patients (460 samples), we dichotomized outcomes based on an IFX trough concentration threshold (≥3 μg/mL). After a systematic evaluation of nine nonlinear ML algorithms, we identified four optimal predictive models. These were subsequently integrated into a soft-voting ensemble classifier to improve predictive performance for individualized IFX monitoring. SHAP analysis was employed to identify key predictors, followed by prospective external validation of dose adjustment strategies.

**Results:**

The ensemble model showed optimal discrimination on the test set (AUC = 0.829, accuracy=0.826, sensitivity=0.778, specificity=0.846, F1 score=0.724) and maintained robust clinical net benefits within a threshold range of 0.48 to 0.62. Five-fold cross-validation confirmed model stability (AUC = 0.850 ± 0.049), and the external validation further demonstrated strong generalizability (AUC = 0.800). SHAP analysis revealed anti-drug antibodies (ADA, 22.8%) and fibrinogen (Fg, 21.4%) as dominant covariates, followed by IFX dose (8.2%). Compared to traditional empirical dosing regimens, the model recommends a more cautious strategy that prioritizes the minimum effective dose to ensure concentrations within the therapeutic window.

**Conclusion:**

We developed and validated an interpretable ensemble model that can dynamically monitor drug concentrations and optimize personalized dosing of IFX therapy in CD patients, demonstrating the potential of an ML-based approach to enhance treatment efficacy and safety.

## 1 Introduction

Infliximab (IFX), a monoclonal antibody targeting tumor necrosis factor-alpha (TNF-α), serves as a key biologic agent for inducing and maintaining remission in Crohn’s disease (CD). Its significant effectiveness in suppressing intestinal inflammation, promoting mucosal healing, and reducing complication risks has established it as a first-line treatment for moderate-to-severe patients (1).

The clinical effectiveness and safety of IFX are closely dependent on trough serum concentrations (2). There is significant variability among individuals, influenced by factors such as body weight, metabolic clearance, and the formation of anti-drug antibodies (ADA), which complicates the ability to maintain precise control over drug exposure. In patients with Crohn’s disease (CD) undergoing IFX therapy, previous research has indicated that maintaining trough concentrations≥3 µg/mL during the maintenance phase is associated with significantly higher rates of clinical remission and mucosal healing (3, 4). Studies indicate that 30%–40% of patients may experience a secondary loss of response due to subtherapeutic drug concentrations and the development of ADA (5). Conversely, supratherapeutic levels (≥7 μg/mL) may increase the risks of infections and malignancies (6, 7). Therefore, the use of therapeutic drug monitoring (TDM) for anti-tumour necrosis factor therapy is currently recommended by several medical societies and international expert groups for CD patients with (6, 8).

Although frequent TDM may benefit CD treatment with IFX, such measurements are not realistic considering the burden on patients and the cost to the healthcare system. Additionally, traditional TDM often depends on empirical dose adjustments and cautious prescribing by clinicians, resulting in interventions only after reduced response or adverse events occur (9). This reactive approach is inherently delayed and struggles to anticipate dynamic concentration changes. Therefore, there is an urgent need for a next-generation TDM strategy that transcends isolated concentration testing. Such an approach should integrate diverse patient characteristics and drug exposure data to establish real-time predictive models (enabling pre-infusion point-of-care predictions) and formulate personalized dosing regimens. This supports the implementation of a proactive “predict-intervene-verify” closed-loop management system. Designed for seamless integration into clinical workflows as an auxiliary tool, it functions as both a precursor and a complement to conventional TDM.

Traditional pharmacokinetic (PK) models employ fixed mathematical structures and predefined physiologic parameters, such as volume of distribution and clearance, to address some aspects of population variability. However, they remain limited in capturing complex non-linear relationships and integrating multimodal clinical covariates, including laboratory parameters, disease activity, and concomitant medications. Artificial intelligence and machine learning (ML) have recently emerged as valuable tools for improving TDM and model-informed precision dosing (10, 11). ML algorithms, including random forests and neural networks, utilize data-driven pattern recognition to dynamically model intricate interactions between patient-specific features and drug exposure, demonstrating particular strength in handling high-dimensional and unstructured data (12, 13). They excel at processing complex and extensive clinical data and have been successfully applied to monitor drug concentrations, including those of voriconazole and sertraline (14, 15). Furthermore, ML models support online learning and real-time updates, enabling dynamic prediction and immediate decision support for dose individualization (16, 17).

This study aims to address the challenge of quantifying the dynamic relationship between concentration and dose during IFX treatment in CD by proposing an integrated ML-based modeling and dose optimization strategy. We will incorporate various clinical data, including patient demographics, laboratory results, immunologic profiles, and treatment history, to develop a dynamic prediction model for IFX trough concentrations. Additionally, we will use reinforcement learning algorithms to identify optimal pathways for adjusting doses to reach target concentration thresholds. The scientific significance of this research includes (1): pioneering the application of ML-driven dynamic covariate modeling to individualize IFX therapy, providing an interpretable tool for addressing complex variability (2); proposing a closed-loop dose optimization system that promotes the advancement of TDM from monitoring of a single concentration to a comprehensive management approach encompassing monitoring, prediction, and intervention; and (3) providing a theoretical foundation and technical framework for developing real-world decision support systems.

## 2 Methods

### 2.1 Patients and data

Serum samples were collected prospectively from CD patients in the Gastroenterology Department of the First Affiliated Hospital of Fujian Medical University between January 2019 and December 2024. All the eligible patients enrolled in the study met the following criteria (1): an established diagnosis of CD (according to the Montreal classification) (2); age ≥6 years (3); patients treated with at least 4 scheduled IFX infusions at weeks 0, 2, and 6, followed by every 8 weeks (q8w) (4); drug levels measured from trough serum samples at week 22 (before the 5th dose). Exclusion criteria included (1) not receiving IFX therapy as prescribed (2); incomplete clinical records (3); other severe systemic diseases. The study was approved by the Ethics Committee of the First Affiliated Hospital of Fujian Medical University in Fuzhou, China (Ethics No.: [2017]120).

Clinical data for CD patients were extracted from the hospital’s Electronic Information System (EIS). These data included IFX trough concentration, dosage regimen, and concomitant medications (azathioprine, mesalazine, and thalidomide), demographic information (gender, age, and body weight), Montreal classification, and laboratory parameters included alanine aminotransferase (ALT), aspartate aminotransferase (AST), creatinine (CREA), albumin (ALB), white blood cell count (WBC), erythrocyte sedimentation rate (ESR), C-reactive protein (CRP), fecal calprotectin (FCP), estimated glomerular filtration rate (eGFR), ADA, Crohn’s disease activity index (CDAI) score, D-Dimer, activated partial thromboplastin time (APTT), and fibrinogen (Fg). In our study, all laboratory measures used as input features in the predictive model were collected at the same time as the corresponding IFX trough concentration measurement, immediately prior to the next scheduled infusion.

### 2.2 Therapeutic drug monitoring of IFX

Venous blood samples were collected from enrolled patients within 30 minutes before the next doses, following at least four previous doses. Serum IFX trough levels and ADA were quantified using enzyme-linked immunosorbent assay kits (ELISA). The detection wavelength for trough concentration was set at 450 nm, with a linear range of 0.01 to45 μg/mL. Both intra-day and inter-day precision is less than 15%.

### 2.3 Data collection and processing

A total of 537 samples were collected according to the inclusion and exclusion criteria. After data cleaning, we assigned values to qualitative variables and standardized formats. Then, missing values with a missing rate greater than 15% in either row or column were removed. In handling missing values, since laboratory indicators such as ALT, AST, D-Dimer, RBC, ALB, and WBC are all continuous numerical variables, and the distributions of these variables do not follow a normal distribution. Median imputation was applied to the remaining missing values in the retained variables, resulting in a final dataset of 460 × 23. In this study, the IFX trough concentration was used as the target variable, with values ≥3 μg/mL assigned 1 and values <3 μg/mL assigned 0, creating a binary classification dataset. To ensure fair model comparison, all samples were assigned identifiers, and the dataset was randomly split into training and test sets in an 8:2 ratio. We applied min-max normalization to the covariates in the training set and used the calculated minimum and maximum parameters to normalize the test set, preventing data leakage [X_scaler = MinMaxScaler(); X_train_scaled = X_scaler.fit_transform(X_train); X_test_scaled = X_scaler.transform(X_test)].

### 2.4 Modeling and validation

#### 2.4.1 Algorithm selection

The linear relationships between IFX trough concentrations and associated covariates were initially assessed (**Supplementary Figure S1**). The analysis revealed no statistically significant linear associations and low correlation coefficients. Given these findings, we employed nine nonlinear ML classification algorithms to develop and screen superior monitoring models for IFX trough concentration prediction. These included Artificial Neural Network (ANN), Decision Tree (DT), Extra Trees (ET), Gradient Boosting Classifier (GBC), K-Nearest Neighbors (KNN), Light Gradient Boosting Machine (LightGBM), Random Forest (RF), eXtreme Gradient Boosting (XGBoost), and Support Vector Machine (SVM). These nonlinear approaches effectively capture complex patterns and nonlinear interactions within the data, thereby providing enhanced modeling capabilities for therapeutic drug monitoring (Python 3.12.7 with scikit-learn package).

#### 2.4.2 Model evaluation metrics

ML evaluation metrics for binary classification models primarily include accuracy, precision, specificity, F1 score, and area under the ROC curve (AUC) (18). Precision is also termed positive predictive value (PPV), and recall is also termed sensitivity. The AUC measures the model’s ability to rank positive versus negative samples. Its values range from 0 to 1, with higher values indicating better model performance (19, 20).

#### 2.4.3 Development and validation of an ensemble model for IFX trough concentration monitoring

To rapidly and accurately identify IFX trough concentration compliance for timely monitoring of CD patients’ medication, we used IFX trough concentration (≥3 μg/ml or <3 μg/ml) as the target variable. The LightGBM classifier was employed to rank feature importance, and key features were selected based on the average AUC value obtained from cross-validation combined with feature combinations. Then, the feature combination with the highest AUC value was selected for model training. Stratified sampling was used to maintain consistent class proportions across the training and testing sets, preventing skewed distributions and ensuring reliable model evaluation (21). Synthetic minority over-sampling technique (SMOTE) was applied only to the training set to address class imbalance. It generates synthetic minority-class samples by interpolation, improving model learning and reducing bias. All preprocessing occurred after train-test splitting to avoid data leakage (22).

To construct and screen for an optimal monitoring model, we employed multiple ML classifiers for training. These included ANN, DT, ET, GBC, KNN, LightGBM, RF, SVM, and XGBoost (23). The optimal model was selected based on its comprehensive performance on the independent test set, evaluated using multiple classification metrics including accuracy, precision, recall, F1-score, and specificity. Hyperparameter optimization was first conducted using GridSearchCV with negative log loss (neg_log_loss) as the objective function to identify the best parameter configuration (24). During the base model screening phase, model evaluation was conducted using two approaches: receiver operating characteristic (ROC) curves and decision curve analysis (DCA). These methods comprehensively assessed the models based on AUC and clinical benefit. Four models performing best in predicting IFX trough concentrations were selected as base models. Employing a Voting Classifier ensemble strategy, we innovatively integrated these four base models (25). By adjusting the weight coefficients of each model, we created an ensemble classifier that combines predictions through soft voting, which averages the predicted probabilities. This approach underwent in-depth visualization analysis. Finally, the predictive performance of the ensemble model was prospectively validated using an independent external dataset. We developed an ensemble model workflow for predicting IFX trough concentrations in CD patients (**Supplementary Figure S2**).

#### 2.4.4 Visualization of ensemble model principal component analysis

First, an ensemble learning framework (Voting Classifier) calculates the classification probabilities of samples. At the same time, PCA is employed to reduce the dimensionality of high-dimensional features. This approach constructs a two-dimensional visualization space with classification probability and the first principal component (PC1), and uses color-scale mapping of actual category labels. It not only quantitatively evaluates the model’s probability calibration across two datasets but also reveals the distribution patterns of samples in the high-dimensional feature space. This provides visual decision support for optimization strategies, including refining classification boundary thresholds and implementing multidimensional feature selection.

#### 2.4.5 Ensemble model SHAP interpretation

The theoretical foundation of the SHapley Additive exPlanations (SHAP) method originates from the SHapley value concept in game theory. Its core mechanism precisely evaluates the incremental impact of various features within ML models on prediction outcomes across multiple collaborative combinations, thereby enabling visual analysis of the model’s decision-making mechanism. This method provides a comprehensive analytical framework that displays the overall influence distribution of all features and allows in-depth analysis of prediction attribution paths for specific samples. This effectively addresses the problem of the complex models’ opaque decision logic. This technical solution is implemented using the Python SHAP library (version 0.47.0) within the Python ecosystem.

### 2.5 Dose optimization strategy

**Figure 1:**
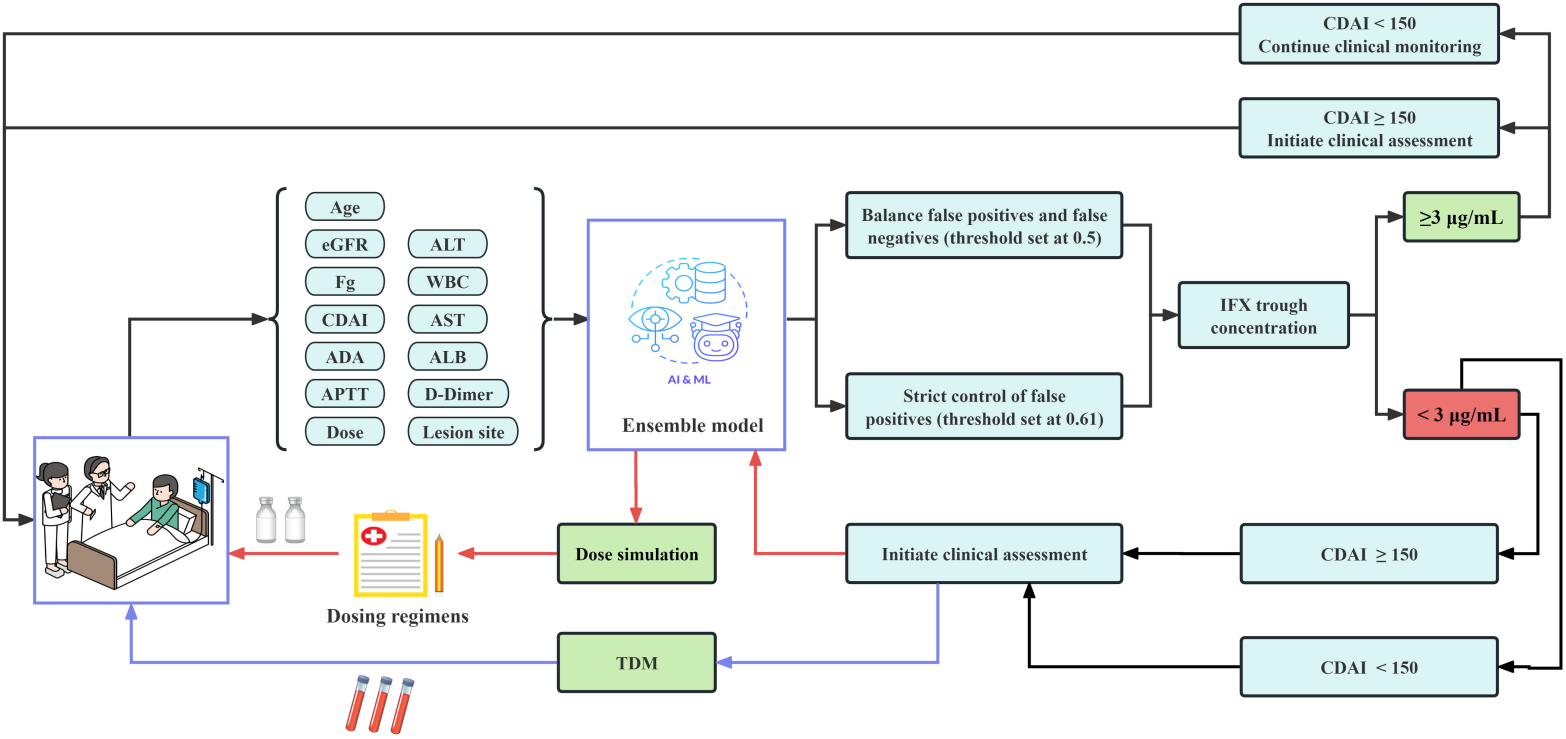
Schematic diagram of dose optimization strategy.

Based on the clinical maintenance therapy requirements for IFX, patient samples with IFX trough concentrations <3 μg/mL were considered. For these samples, the following key clinical parameters shall remain constant: Age, ADA, Fg, eGFR, ALB, CDAI, ALT, AST, APTT, D-Dimer, WBC, and Lesion site. We implemented a simulation protocol with 10 mg dose increments under an initial ensemble model threshold of 0.50. When the ensemble model first predicts the IFX trough concentration reaching the therapeutic window (≥3 μg/mL), we recommend that dose, with decision-making further informed by CDAI scores. Additionally, in clinical scenarios requiring strict control of false positives, we set the ensemble model threshold to 0.61 to better identify samples below the therapeutic level (<3 μg/mL). For these samples, decisions can be made based on CDAI scores and TDM results (**Figure 1**). To validate its clinical feasibility, we specifically selected eight clinical samples at risk of inadequate drug exposure (<3 μg/mL) for retrospective analysis. These data included patient baseline characteristics and conventional empirical dose adjustment protocols.

### 2.6 Statistical analysis

Data analysis was performed using IBM SPSS Statistics 25.0. Categorical variables are presented as frequencies (n, %), and differences between groups were assessed using chi-square tests. Continuous variables are described as mean ± standard deviation (SD). For normally distributed data, intergroup comparisons were employed using independent samples t-tests; for non-normally distributed data, the Mann-Whitney U test was used. Correlations between variables were assessed using Spearman’s rank correlation coefficient, and p-values less than 0.05 were considered statistically significant.

## 3 Results

### 3.1 Baseline information of CD

This study used clinical data from 274 CD patients to create a dataset containing 460 IFX trough concentration measurements (460×23) (**Table 1**). We used the steady-state trough concentration threshold (≥3 μg/mL) as the categorization criterion. Then, features were selected through a systematic preprocessing workflow. This process produced a refined dataset of 460 samples with 14 optimized features for modeling. The dataset was randomly divided into training and test sets at an 8:2 ratio, using a fixed random seed to ensure experimental reproducibility (**Supplementary Figure S3**).

**Table 1:** Baseline characteristics of CD in the ensemble model.

Variables	Values		
	Train (n=368)	Test (n=92)	*P value*
Gender, n (%)			0.731^a^
female	127 (34.5)	30 (32.6)	
male	241 (65.5)	62 (67.4)	
*Age (years)	25.95 ± 8.99	25.83 ± 9.37	0.688^b^
*IFX trough concentration, n (%)			0.918^a^
≥3 μg/mL	262 (71.2)	65 (70.7)	
<3 μg/mL	106 (28.8)	27 (29.3)	
*IFX dose (mg/kg)	5.70 ± 1.05	5.64 ± 1.00	0.826^b^
*ADA, n (%)			0.044^a^
Negative	299 (81.3)	66 (71.7)	
Positive	69 (18.7)	26 (28.3)	
*Fg (g/L)	2.87 ± 0.97	2.98 ± 1.14	0.736^b^
*eGFR (mL/min)	125.57 ± 13.64	124.03 ± 15.15	0.413^b^
*ALB (g/L)	43.72 ± 3.42	43.64 ± 3.91	0.801^b^
*CDAI	35.74 ± 49.22	42.08 ± 53.90	0.212^b^
*ALT (U/L)	18.57 ± 15.38	18.39 ± 11.65	0.586^b^
*AST (U/L)	20.27 ± 7.71	19.46 ± 6.12	0.570^b^
*APTT (S)	30.65 ± 5.24	30.60 ± 4.38	0.566^b^
*D-Dimer (μg/mL)	0.22 ± 0.27	0.22 ± 0.21	0.229^b^
*WBC (10^9/L)	5.97 ± 1.67	6.21 ± 2.04	0.431^b^
Fecal calprotectin, n (%)			0.132^a^
Negative	65 (17.7)	21 (22.8)	
Weak Positive	59 (16.0)	18 (19.6)	
Positive	244 (66.3)	53 (57.6)	
Montreal age, n (%)			0.709^a^
≤16	91 (24.7)	25 (27.2)	
17-40	262 (71.2)	63 (68.5)	
>40	15 (4.1)	4 (4.3)	
*Lesion site, n (%)			0.108^a^
L1	8 (2.2)	3 (3.3)	
L2	10 (2.7)	5 (5.4)	
L3	181 (49.2)	31 (33.7)	
L1+L4	16 (4.3)	5 (5.4)	
L2+L4	5 (1.4)	0 (0.0)	
L3+L4	148 (40.2)	48 (52.2)	
Behavior_B, n (%)			0.381^a^
B1	196 (53.3)	53 (57.6)	
B2	135 (36.7)	31 (33.7)	
B3	21 (5.7)	6 (6.5)	
B2+B3	16 (4.3)	2 (2.2)	
Behavior_P, n (%)			0.162^a^
NO	131 (35.6)	40 (43.5)	
YES	237 (64.4)	52 (56.5)	
Azathioprine, n (%)			0.091^a^
NO	225 (61.1)	65 (70.7)	
YES	143 (38.9)	27 (29.3)	
Mesalazine, n (%)			0.019^a^
NO	332 (90.2)	75 (81.5)	
YES	36 (9.8)	17 (18.5)	
Hormone, n (%)			0.856^a^
NO	301 (81.8)	76 (82.6)	
YES	67 (18.2)	16 (17.4)	
Thalidomide, n (%)			0.193^a^
NO	346 (94.0)	83 (90.2)	
YES	22 (6.0)	9 (9.8)	

a Chi-squared test.

b Mann-Whitney U test.

*Variables of the ensemble model

### 3.2 Development and validation of IFX trough concentration monitoring model

#### 3.2.1 Covariate screening and ML algorithm selection

**Figure 2:**
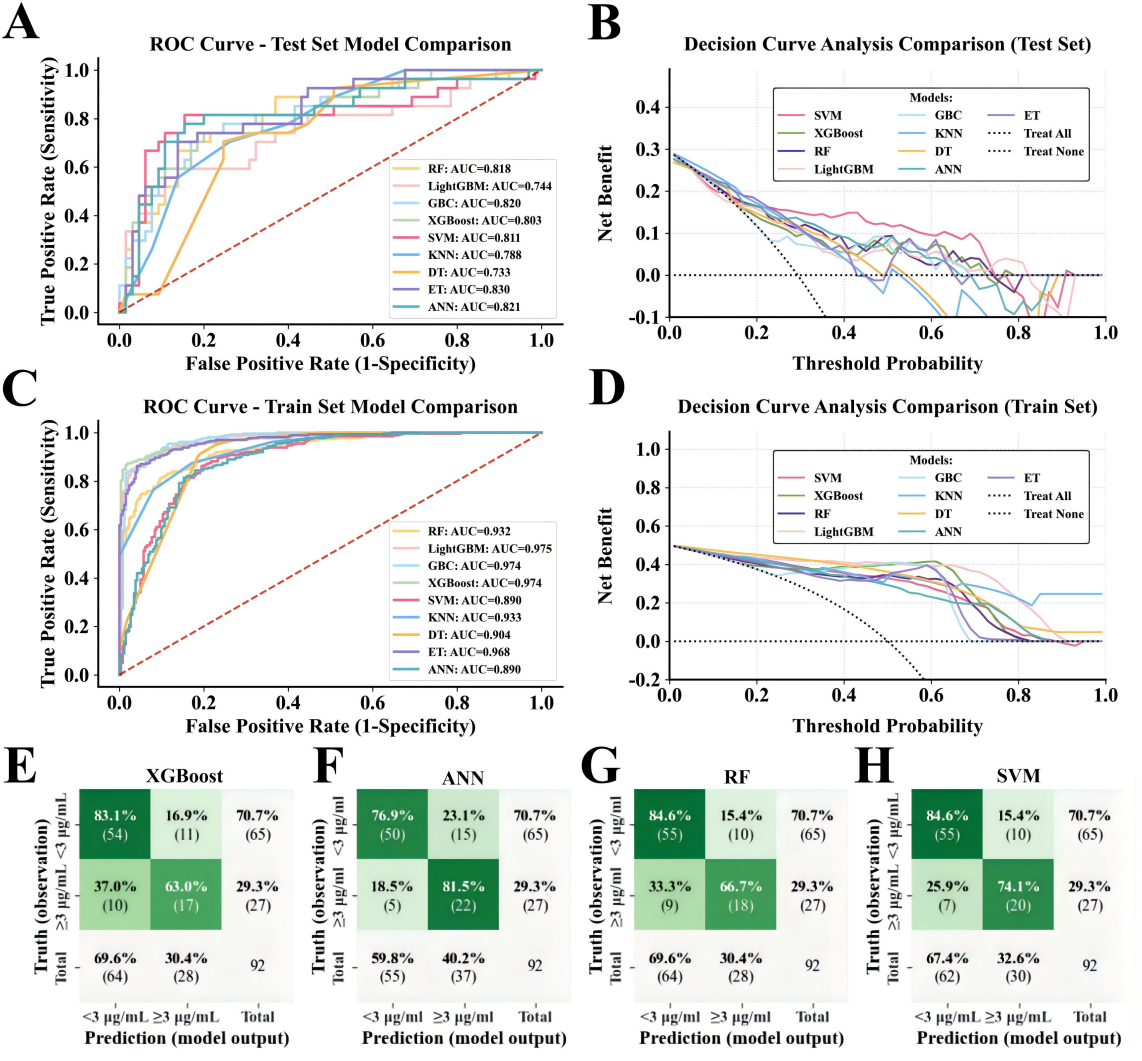
Comparative evaluation of nine ML algorithms. **(A-C)** show the ROC curves and AUC values of IFX on the test and training sets, respectively; the red dashed line represents the random classification baseline. **(B, D)** demonstrate the DCA performance comparison of IFX on the test and training sets. **(E-H)** present the confusion matrices of the four base models (XGBoost, ANN, RF, and SVM) on the test set.

We identified the optimal feature subset through rigorous feature selection, applied stratified sampling and SMOTE to address class imbalance, and systematically evaluated nine machine learning algorithms (**Figures 2A-D**). XGBoost, ANN, RF, SVM, GBC, and ET achieved high discriminative power with AUCs above 0.800 in the independent test set. Although GBC and ET showed certain predictive performance, DCA revealed they had significantly lower net clinical benefit than other models; therefore, based on both predictive performance and clinical benefit, XGBoost, ANN, RF, and SVM were selected as the core algorithms for subsequent ensemble modeling (**Supplementary Figure S2**).

#### 3.2.2 Ensemble model construction

**Figure 3:**
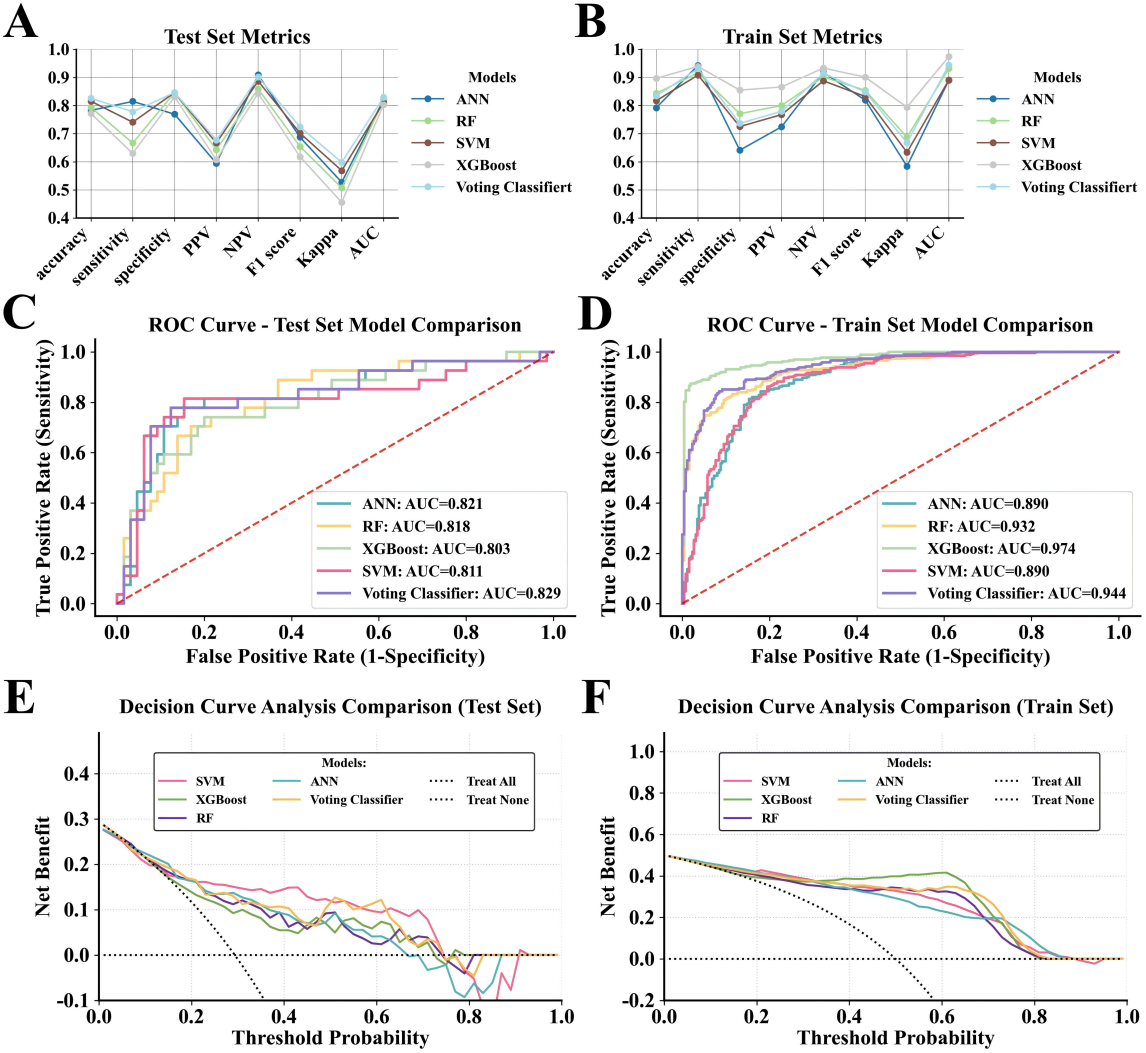
Evaluation of the ensemble models. **(A, B)** show the comparison of prediction performance metrics between ensemble and baseline models on the training and testing datasets. (**C, D)** display the ROC curves for ensemble models and baseline models on the training and testing datasets. **(E, F)** present the comparison of clinical benefit in DCA between ensemble models and baseline models on the training and testing datasets.

**Figure 4:**
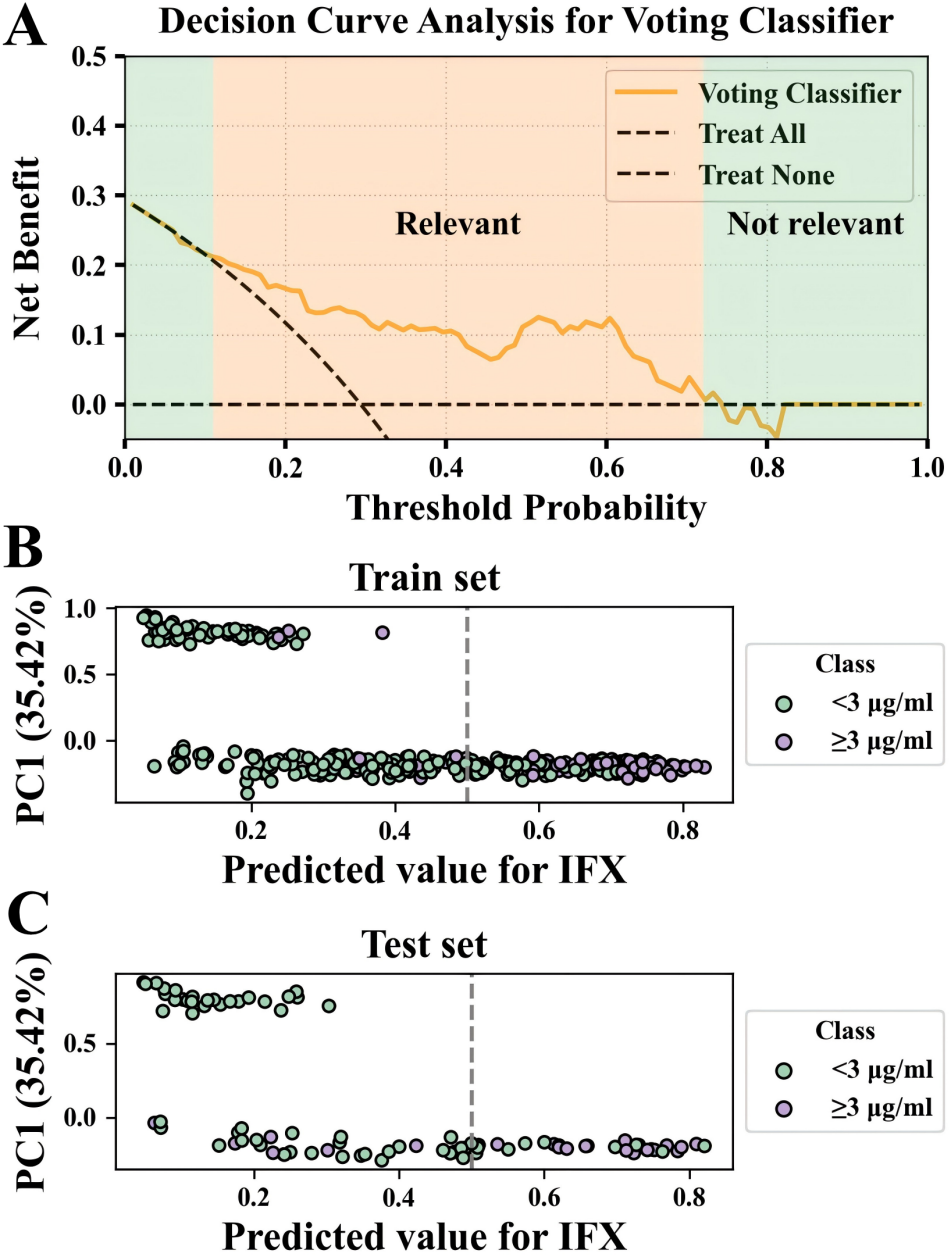
DCA and PCA visualization of the ensemble model. **(A)** DCA shows the clinical net benefit of the ensemble model on the test set; the orange-shaded region (risk threshold interval: 0.11-0.72) indicates thresholds where predictions significantly improve clinical decisions. **(B, C)** X-axis predicts probability of IFX trough concentration ≥3 μg/mL; gray dashed line marks the classification threshold (0.50). Y-axis explains 35.42% of the original data variance and reflects projections along the direction of maximal variability.

This study used a Voting Classifier ensemble strategy to integrate four heterogeneous modeling approaches (XGBoost, ANN, RF, and SVM) through novel fusion. A soft voting mechanism (probability averaging) assigned equal weights (1:1:1:1) to base models. Results showed that the ensemble model achieved better predictive performance using the test set. (**Figure 3A**) In the clinical utility analysis, the ensemble model exhibited robust advantage in net benefit values across the risk threshold range of 0.48-0.62, outperforming all base models in DCA (**Figure 3E**). To validate stability, stratified five-fold cross-validation yielded a mean AUC of 0.850 ± 0.049 (SD), indicating strong internal validity. Notably, the model retained high predictive performance (AUC = 0.800) in an independent external validation cohort (**Table 2**), confirming its clinical generalizability. Further DCA analysis on the test set showed that within the 0.11-0.72 risk threshold range (orange region), the ensemble model’s predictions significantly enhanced clinical decision-making (**Figure 4A**).

**Table 2:** Evaluation of the ensemble model predictive performance (threshold = 0.50).

Group	Accuracy	PPV	Sensitivity	Specificity	F1-score	AUC
Train	0.834	0.780	0.931	0.737	0.849	0.944
Test	0.826	0.677	0.778	0.846	0.724	0.829
Mean CV Score	0.771 ± 0.071	0.726 ± 0.060	0.870 ± 0.105	0.672 ± 0.069	0.790 ± 0.073	0.850 ± 0.049
Validation	0.714	0.692	0.600	0.800	0.643	0.800

PPV, Positive predictive value; AUC, area under the ROC curve.

Mean CV score, Mean 5-fold cross-validation score.

Validation, 35 in-hospital data samples.

#### 3.2.3 PCA visualization and performance optimization of the ensemble model

The ensemble model first computed classification probabilities for all samples. A PCA transformation was fitted on the training set to extract PC1, which accounted for 35.42% of the original variance and captured the direction of maximal variability in the data. The trained PCA model was then consistently applied to transform features in both training and test sets. As shown in **Figure 4**, the model demonstrated clinically significant separation between green (subtherapeutic, <3 μg/mL) and purple (therapeutic, ≥3 μg/mL) clusters at the 0.50 classification threshold, confirming a stable decision boundary across datasets. This probabilistic-spatial segregation indicates that the selected threshold optimally balances interpretability, aligning with clinical decision protocols. It also effectively controls misclassification risk for borderline samples.

Based on DCA curve and PCA visualization results (**Figure 4**), we recalibrated the decision threshold of the ensemble model to 0.61. At this threshold, the AUC remained unchanged, while sensitivity decreased slightly. However, other core metrics were significantly optimized: PPV and specificity substantially improved, false positive classifications (≥3 μg/mL) decreased markedly, and the accuracy of identifying negative samples (<3 μg/mL) was enhanced. This setting demonstrated strong consistency and robustness in both the test and external validation sets (**Table 3**). Clinically, a high false positive rate could overestimate IFX therapeutic concentrations, potentially leading to insufficient dosing or treatment failure, which in turn increased medical risks. In contrast, the 0.61 threshold provides greater clinical utility and safety when strict false positive control is needed.

**Table 3:** Optimized prediction performance of the ensemble model (Threshold = 0.61).

Group	Accuracy	PPV	Sensitivity	Specificity	F1-score	AUC
Train	0.876	0.896	0.851	0.901	0.873	0.944
Test	0.859	0.792	0.704	0.923	0.745	0.829
Validation	0.771	0.818	0.600	0.900	0.692	0.800

PPV, Positive predictive value; AUC, area under the ROC curve.

Mean CV score, Mean five-fold cross-validation score.

Validation, 35 in-hospital data samples.

#### 3.2.4 SHAP interpretation of the ensemble model

**Figure 5:**
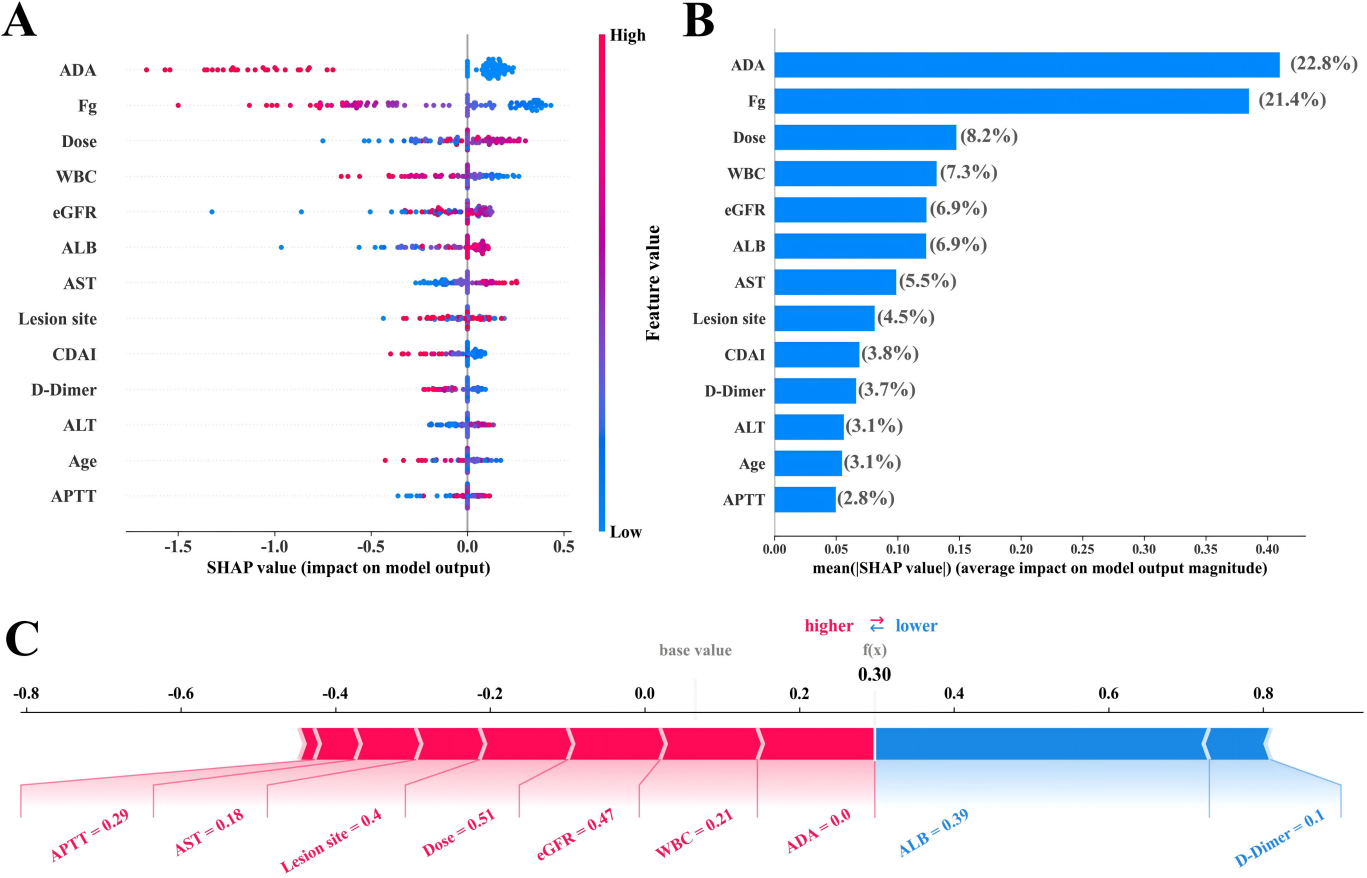
SHAP-based interpretability of feature importance and force plots for the ensemble model. **(A)** The feature contribution ranking for the IFX trough concentration binary classification task is ordered by descending mean absolute SHAP values. Percentages indicate the relative contribution of each feature to model output. **(B)** Beeswarm plot: Each point represents the SHAP value of a feature for an individual sample. Features are ordered vertically by global importance, from highest (top) to lowest (bottom). The horizontal axis shows SHAP values, where positive values (right) indicate contributions toward a higher predicted risk class, and negative values (left) indicate contributions toward a lower risk class. Color intensity reflects feature magnitude (red: high, blue: low). **(C)** SHAP force plot for Sample No. 1 in the test set, illustrating the directional impact of key features on the predicted IFX trough concentration category.

**Figure 6:**
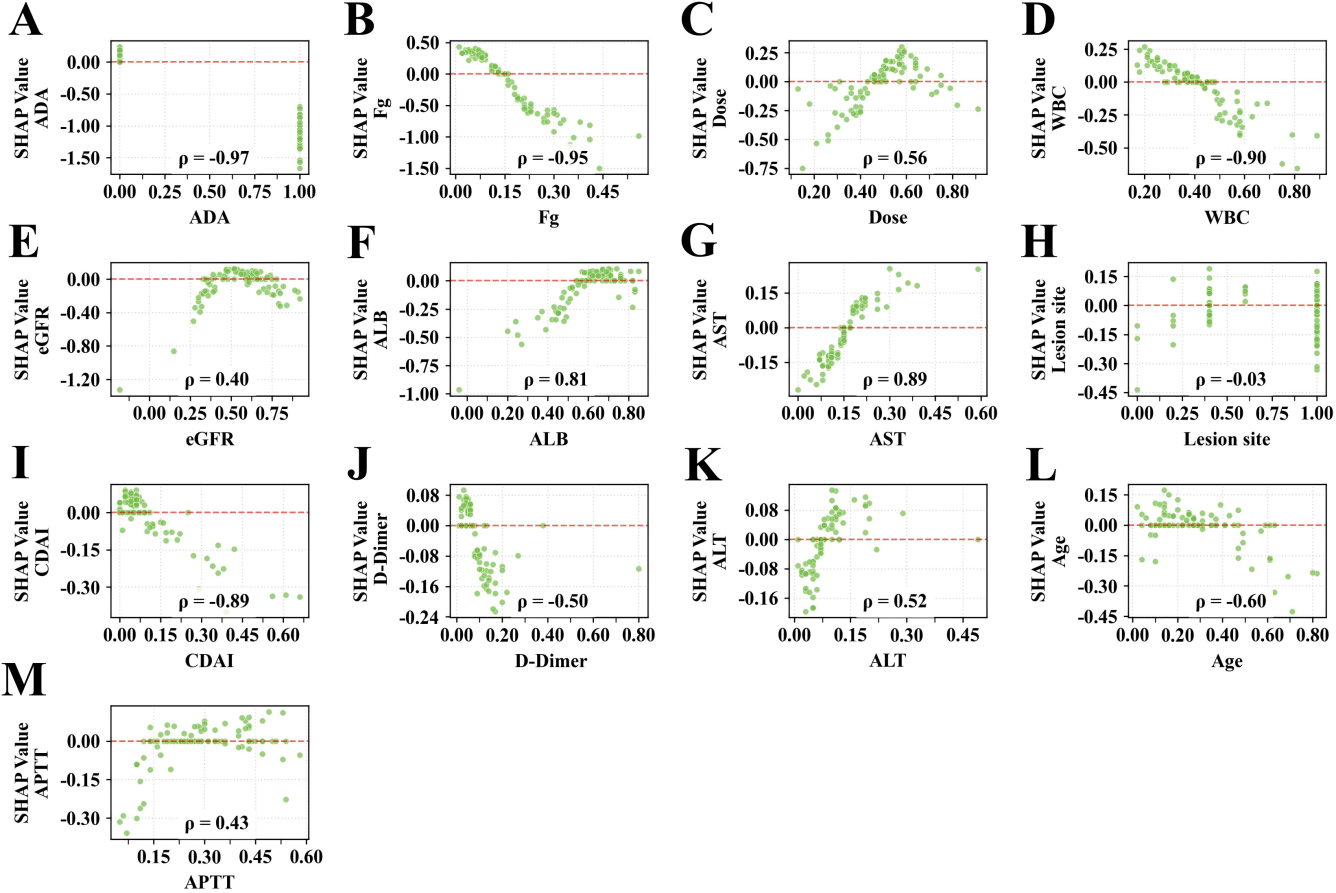
SHAP-based interpretation of feature importance and dependency relationships for the ensemble model **(A–M)**. Importance ranking of all covariates in the ensemble model. SHAP dependency plots further quantify the contribution of features to the target classification outcome.

SHAP visualization analysis of the final ensemble Voting Classifier model revealed that ADA (22.8%) and Fg (21.4%) were the two most important features, followed by IFX dose (8.2%), WBC (7.3%), eGFR (6.9%), and ALB (6.9%) (**Supplementary Figure S3** and **Figure 5**). Notably, ADA, Fg, WBC, and CDAI showed significant negative correlations with IFX trough concentrations. Conversely, IFX dose, ALB, and AST were positively correlated (**Figure 6**).

### 3.3 Validation of dose optimization strategy

We developed a data-driven clinical personalized dosing strategy through a concentration-dose optimization clinical translation framework. This framework dynamically adjusts model thresholds according to different clinical scenarios and generates real-time optimal dosing regimens (**Figure 1**). Clinical validation confirmed its effectiveness, demonstrating the integrated model’s excellent generalization performance and its ability to simulate dosing. Key findings indicate that, compared to traditional empirical dosing protocols, the doses recommended by the model follow a more prudent approach. They prioritized the minimum effective dose principle while ensuring serum drug concentrations reach the therapeutic window, thereby optimizing clinical safety margins (**Table 4**).

**Table 4:** Comparison of dose simulation based on the IFX ensemble model and traditional empirical dosing methods.

No.	Dose adjustment method	Pre-adjustment dose (mg)	Dose (mg/kg)	Pre-adjustment concentration (μg/mL)	Post-adjustment dose (mg)	Dose (mg/kg)	Post-adjustment concentration (μg/mL)
1	Empirical therapy	300	4.62	< 3	400	6.15	≥3
	Ensemble model	300	4.62	< 3	340	5.23	≥3
2	Empirical therapy	300	5.17	< 3	400	6.90	≥3
	Ensemble model	300	5.17	< 3	330	5.69	≥3
3	Empirical therapy	300	4.29	< 3	400	5.71	≥3
	Ensemble model	300	4.29	< 3	350	5.00	≥3
4	Empirical therapy	200	3.08	< 3	400	6.15	≥3
	Ensemble model	200	3.08	< 3	260	4.00	≥3
5	Empirical therapy	400	5.13	< 3	400	5.13	≥3
	Ensemble model	400	5.13	< 3	460	5.90	≥3
6	Empirical therapy	200	4.08	< 3	300	6.12	≥3
	Ensemble model	200	4.08	< 3	230	4.69	≥3
7	Empirical therapy	300	4.84	< 3	406	6.55	≥3
	Ensemble model	300	4.84	< 3	340	5.48	≥3
8	Empirical therapy	200	3.08	< 3	300	4.62	≥3
	Ensemble model	200	3.08	< 3	230	3.54	≥3

## 4 Discussion

This study addresses a critical challenge in CD management by optimizing IFX dosing using a data-driven precision monitoring approach, effectively bridging the gap between TDM and clinical decision-making. Although traditional TDM is fundamental to IFX therapy, the reliance on empirical dose adjustments often results in delayed interventions and fails to consider key clinical variables, which can lead to suboptimal therapeutic responses, inadequate trough concentrations, and unnecessary drug exposure. To address these issues, we developed a ML-based ensemble framework that provides a scalable and systematic strategy for dose optimization. By integrating diverse nonlinear ML algorithms (XGBoost, ANN, RF, and SVM) into a soft-voting classifier, our model addresses the inability of single-model approaches to capture complex biological interactions and demonstrates robust performance stability and generalization capability, supporting its potential for clinical application (**Figure 3**). Notably, the model-guided dose optimization reduced dependence on empirical protocols while achieving comparable or superior therapeutic outcomes with lower drug doses, potentially minimizing risks of immunogenicity and adverse events (**Table 4**).

SHAP interpretability analysis identified key predictors influencing IFX trough concentrations and their direction of effects, including ADA, Fg, dose, ALB, WBC, et al, and most of them are routine test indexes, ensuring simplicity, objectivity, low invasiveness, quick obtainability, and cost-effectiveness. Additionally, easily obtainable factors such as eGFR, AST, Montreal classification, CDAI score, and age also contribute to the model’s efficacy (**Figure 6**).

We observed that ADA (ranked first, 22.8%) was the most influential factor and showed a strong negative correlation with IFX trough concentration (ρ = -0.97). Consistent with previous studies, ADA formation accelerates drug clearance (CL) and increases the risk of loss of response (26). A prospective observational study found that 75% of patients developed ADAs to IFX by week 22, increasing to 90% within the first 12 months of therapy (27). Brandse et al. demonstrated that insufficient exposure to IFX, defined by the time IFX concentrations fell below a trough level of 3 μg/mL during a dose interval, was the most predictive factor for developing ADAs (28). The presence of immunogenicity against IFX may reduce its exposure through the formation of immune complexes that accelerate CL via the reticuloendothelial system and/or impair its efficacy by blocking antigen binding, thereby increasing the risk of treatment failure (29). Therefore, our findings further underscore the importance of monitoring ADA as a primary early-warning indicator during IFX therapy.

The management of CD, which is characterized by persistent inflammation that can fluctuate over time, leading to periods of active disease and remission, has traditionally depended on endoscopic assessment for evaluating disease activity and treatment efficacy (30). While endoscopy remains the gold standard, its invasiveness, cost, and patient discomfort underscore the need for alternative methods. The search for reliable, noninvasive biomarkers that can predict endoscopic improvement has become a cornerstone of contemporary research in CD management (31).

Fg (ranked second, 21.4%), an acute-phase reactant often elevated during CD flares (32, 33), proved to be a useful biomarker in our analysis, demonstrating a significant negative correlation with IFX concentration (ρ= -0.95). Elevated Fg may reflect a high inflammatory burden. A high-inflammatory state may increase IFX clearance through various pathways (e.g., increased vascular permeability, accelerated monoclonal antibody clearance via the reticuloendothelial system). Additionally, high inflammation can predispose to immunogenicity (ADA formation) and lead to lower trough concentrations, which may necessitate higher IFX exposure, such as intestinal mucosal damage or systemic inflammation. Supporting this, the study by Wang et al. (34), represents a significant step forward. By identifying changes in ALB and Fg as effective predictors of endoscopic improvement in CD. ALB, a protein indicative of nutritional status and liver function, also reflects systemic inflammation (32). Low levels of ALB have been linked to increased disease activity and poor clinical outcomes in patients with CD (35, 36).

In our study, ALB (ranked sixth, 6.9%) showed a positive correlation with IFX concentration (ρ= 0.82). Fasanmade et al. analyzed data from two clinical trials to evaluate the relationship between IFX PKs and serum ALB (37). Low ALB levels, reflecting systemic inflammation or malnutrition, are associated with reduced IFX exposure and poorer clinical outcomes. Previous studies have reported that patients with higher ALB levels exhibit higher IFX trough concentrations and improved response rates. This relationship may be attributed to shared elimination pathways and the role of ALB in maintaining IgG stability. Additionally, as IgG antibodies are proteins, they rely on energy derived from catabolism. In states of ALB deficiency, the body may accelerate the IgG metabolism to meet energy demands, thereby reducing serum IFX concentration (38, 39).

The primary advantage of using biomarkers such as ALB and Fg is that they can serve as noninvasive tools, offering both convenience and accuracy, with the potential to revolutionize the way CD is monitored. Blood tests are simple, affordable, and suitable for regular monitoring, offering valuable insights into a patient’s condition without the discomfort and risks of invasive exams. Furthermore, they can be easily integrated into routine clinical practice, thereby improving the efficiency of patient management.

IFX dose (mg/kg, ranked third, 8.2%) was positively correlated with trough concentration (ρ = 0.56), consistent with its linear PKs. Our cohort predominantly received standard weight-based dosing (5mg/kg), reducing its variance and thus its relative importance compared to more variable factors like ADA and Fg. Therefore, its relatively low contribution suggests that dose adjustment alone may be insufficient for precise concentration control. Notably, only 28.9% of patients on IFX maintenance therapy in this study achieved therapeutic trough levels (≥3.0 μg/mL), highlighting the need for comprehensive TDM strategies that incorporate immune status (e.g., ADA) and inflammatory markers (e.g., Fg, WBC).

We observed a negative correlation between WBC (ranked fourth, 7.3%) and IFX concentration (ρ = -0.90). Persistently elevated WBC may indicate subtherapeutic drug exposure and active inflammation. Previous studies have suggested that WBC and PLTs are highly associated with disease severity in pediatric ulcerative colitis. Increased WBCs, specifically neutrophils, may contribute to IFX clearance via Fcγ receptor-mediated elimination or protease activity (40–42). Higher disease activity amplifies TNF-α production, leading to accelerated IFX clearance through target-mediated drug disposition (42–44). These mechanisms support the relevance of WBC as a practical and predictive marker for optimizing IFX therapy.

Calprotectin, a clinically widely used fecal biomarker, serves as a marker for neutrophil migration in the gastrointestinal tract and demonstrates higher specificity compared to other commonly used inflammatory markers like C-reactive protein (45, 46). FCP levels correlate closely with endoscopic and histopathological indicators of disease activity and relapse (47, 48). However, FCP has several limitations. Its sensitivity and specificity depend on the location of inflammation. Several studies have reported lower specificity in CD patients compared to those ulcerative colitis patients (49). This may explain its limited utility in small bowel disease, and consequently, its role in predicting drug exposure in CD patients remains controversial (50).

Our analysis suggests that incorporating eGFR and ALT monitoring alongside TDM could improve the identification of patients at risk of subtherapeutic IFX exposure. Impaired renal function may promote fluid overload and dysregulated cytokine profiles, potentially accelerating IFX clearance via proteolytic degradation and immune-mediated pathways. Similarly, elevated ALT levels often indicative of systemic inflammation or hepatic stress, may compromise ALB synthesis, disrupt FcRn-mediated IgG recycling, and enhance cytokine-driven clearance mechanisms, collectively contributing to reduced IFX trough concentrations.

The CDAI is a composite instrument that incorporates patient-reported items (abdominal pain and including general well-being), physician-reported items (abdominal mass and extraintestinal findings) and laboratory findings (i.e. haematocrit). For decades, the CDAI has been regarded as a valuable tool for measuring the clinical activity of CD. Clinical remission was defined as a CDAI score of <150 (51).

Our study proposes a data-driven concentration-dose optimization framework for clinical translation. This framework targets patients undergoing IFX maintenance therapy who have subtherapeutic trough concentrations (<3 μg/mL) (**Figure 1**). Integrating a dynamic dose adjustment model with CDAI, our study provides a new approach for personalized treatment. Unlike traditional empirical dose adjustments, which often depend on clinicians’ subjective judgments and may result in underexposure or overexposure. This study incorporated multidimensional parameters, including patient age, ADA, biochemical indicators like ALB and eGFR, and inflammatory activity measured by CDAI, to construct a dynamic dose simulation model. The results demonstrated that the ensemble model-recommended dosing regimen better adhered to the “minimum effective dose” principle. It achieved target trough concentrations (≥3 μg/mL) while significantly reducing the required drug dosage (**Table 4**). This approach aligns with the recent emphasis on precision strategies combining TDM and PK modeling (52).

The innovation of this study lies in its introduction of a dynamic threshold mechanism. It allows adjustment of model sensitivity and specificity based on different clinical scenarios such as strict control of false positives, thereby enhancing decision-making flexibility. Two key thresholds were set (0.50 vs. 0.61), corresponding to conventional scenarios and scenarios requiring strict control of false positives, respectively. When the threshold was raised to 0.61, the model prioritized identifying insufficient trough concentrations as negative samples, reducing unnecessary interventions in low-risk patients. This strategy helps avoid unnecessary dose escalations. Previous studies often used fixed thresholds, which resulted in delayed treatment for some patients due to false-positive results (53). In contrast, the dynamic threshold adjustment function of this framework addresses this gap and improves clinical applicability.

The model integrates dose recommendations with CDAI scores to enhance the combined evaluation of efficacy and safety. For example, in patients with high CDAI scores, which indicate active disease, the model suggests escalating the dose to quickly manage inflammation. Conversely, if the CDAI is low but trough concentrations are still below therapeutic levels, potential causes related to immunogenicity, such as elevated ADA, or metabolic irregularities should be considered. This comprehensive decision-making model surpasses traditional one-dimensional TDM strategies and is in line with the 2023 ECCO guideline recommendation for personalized treatment that incorporates clinical data, biomarker information, and drug concentration data (54).

Despite these promising advancements, the study has limitations. Firstly, this was a prospective study with a relatively small sample size and short follow-up, which may introduce selection bias and constrain generalizability. Secondly, objective measures of treatment response, including endoscopic, histologic, or pharmacogenetic data, were not incorporated due to insufficient availability. Thirdly, the framework’s real-time simulation capability depends on high-quality input data, including timely CDAI assessments, which presents challenges for integrating real-world data. The model’s performance may vary due to differences in laboratory testing methods. The most prudent strategy is to perform slight calibration or validation of the model using local data from the target hospital before deployment. Future work should focus on developing embedded clinical decision support systems (CDSS) integrated with electronic medical record systems to enable automated dose recommendations. Additionally, multicenter randomized prospective studies with larger sample sizes, longer follow-up periods, and more comprehensive evaluations are needed to validate the model’s effects on long-term outcomes, safety, and cost-effectiveness, ultimately fostering innovation in clinical practice.

In summary, our study has successfully constructed a predictive ensemble model using an ML approach to monitor drug concentrations dynamically and optimize individualized treatment of IFX. This model was designed to be integrated into clinical workflows as an auxiliary tool (decision-support aid), serving as both a precursor and a complement to conventional TDM. This model was intended to supplement, not replace, clinical judgment. When the model’s recommended dosage conflicts with the physician’s assessment, clinicians should prioritize a comprehensive evaluation that takes into account the patient’s comorbidities, concurrent infections, symptom presentation, and personal preferences. This enables clinicians to identify high-risk patients for preemptive dose optimization, thereby potentially enhancing long-term treatment outcomes.

## Data Availability

The original contributions presented in the study are included in the article/**Supplementary Material**. Further inquiries can be directed to the corresponding authors.
