# Supplementary material for: Concentration monitoring and dose optimization for infliximab in Crohn’s disease patients: a machine learning-based covariate ensemble model
Source: Front Immunol. 2025 Dec 8;16:1715241. doi: 10.3389/fimmu.2025.1715241 (PMC12719469; doi:10.3389/fimmu.2025.1715241)
Supplement: Supplementary file 1 [file Table1.docx]

**Concentration monitoring and dose optimization for infliximab in Crohn's Disease patients: a machine learning-based covariate ensemble model**

Yuewen Chen^a, b,1^, Shoutian Zhang^a, b,1^, Si Chen^c^, Shaojun Jiang^a, b^, Shuifang Zhou^a, b^, Jing Liu^a, b^, Zhoujie Liu^a, b^, Rongfang Lin^a, b,#^, Jianwen Xu^a,b,#^

**Supporting data** (Figures S1-S3)


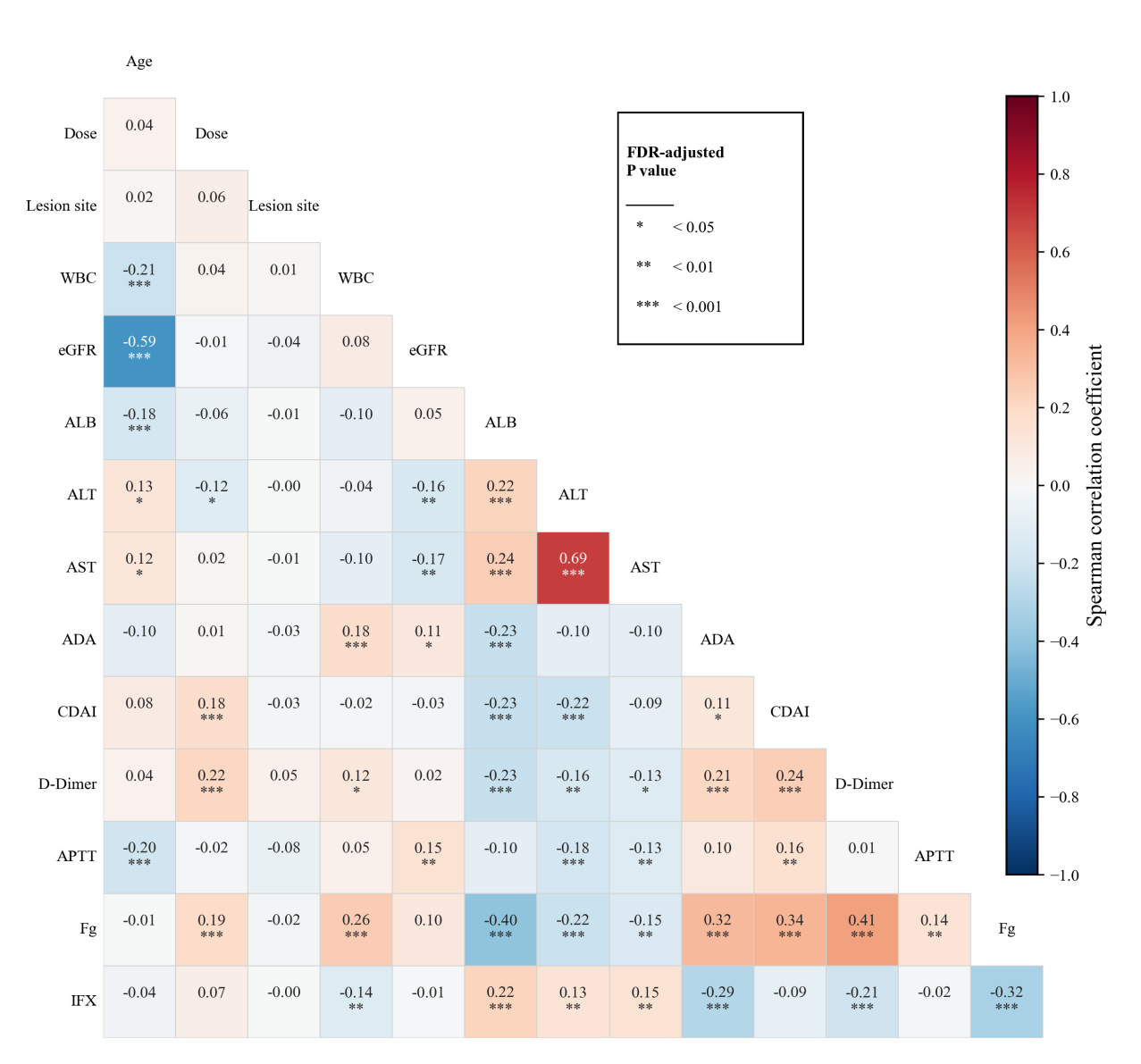


FIGURE S1 Spearman's rank correlation matrix. The lower triangular section displays correlation coefficients with false discovery rate (FDR)-adjusted significance markers. A red-blue color gradient indicates the strength and direction of correlations (red: positive; blue: negative).


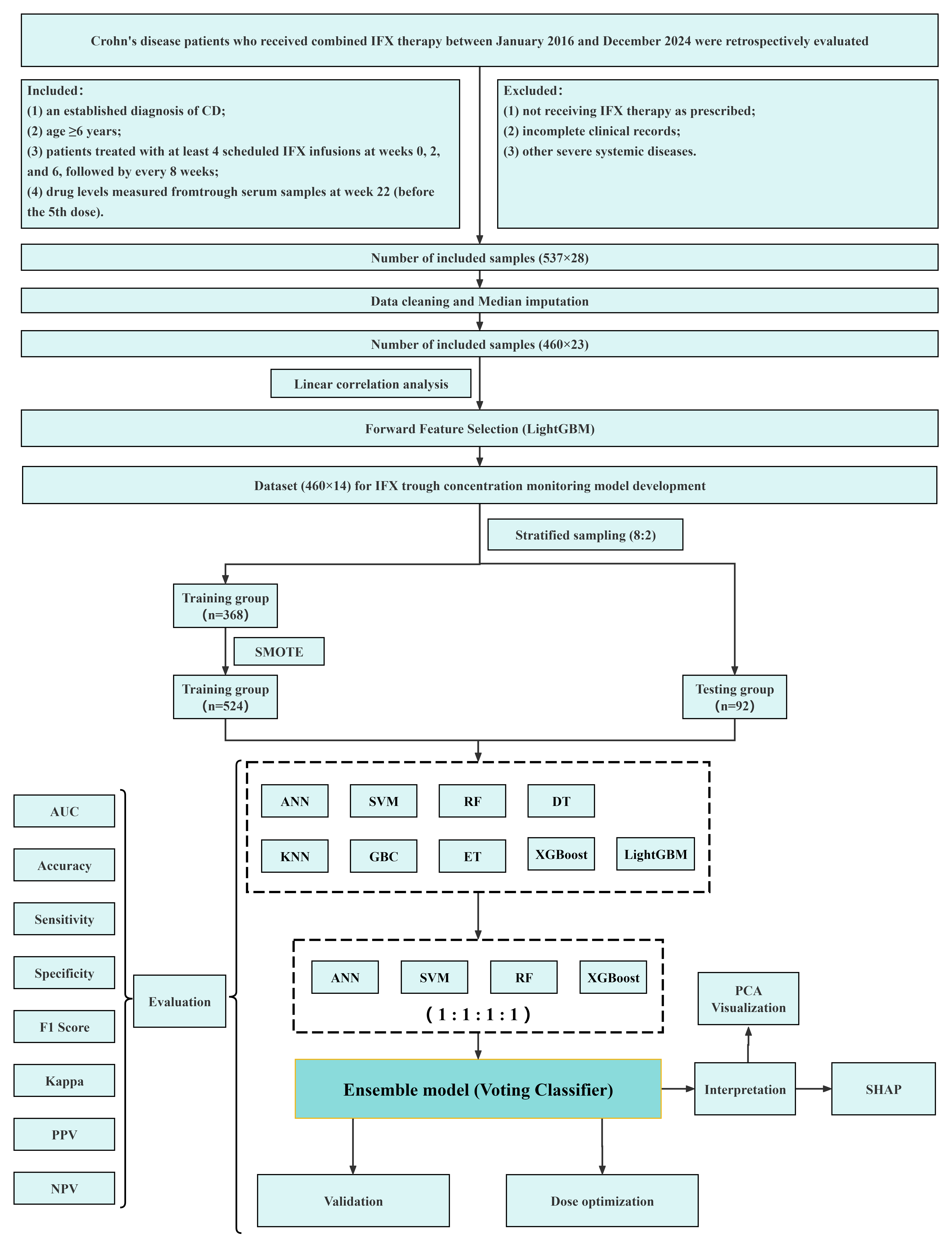


FIGURE S2 Flowchart illustrating the development of an ensemble model for predicting infliximab trough concentrations in patients with Crohn's disease.


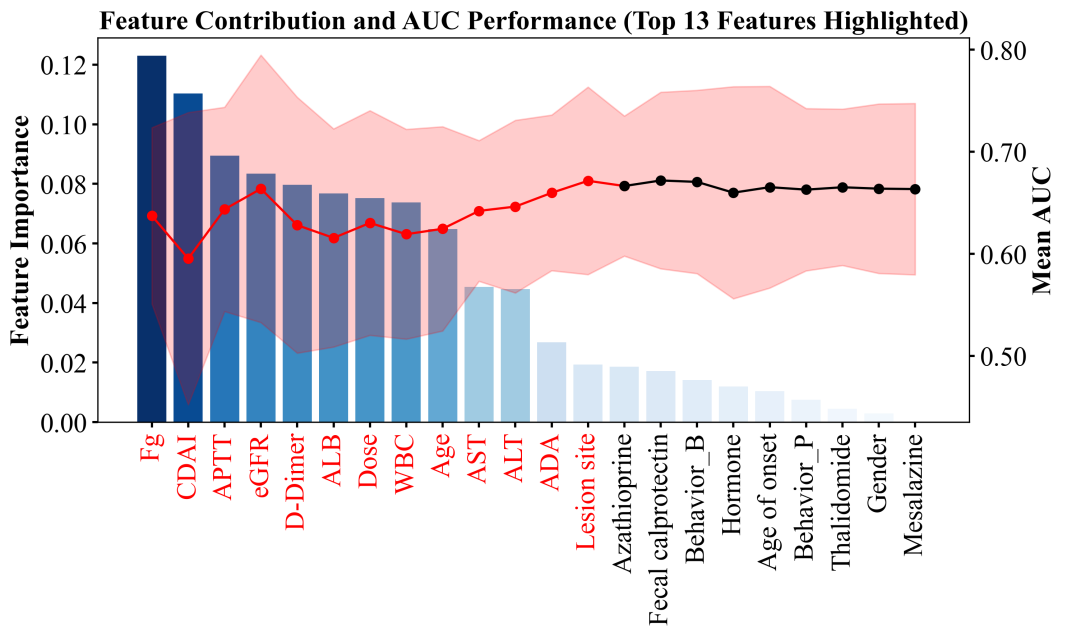


FIGURE S3 Schematic of the feature selection workflow. The optimal feature subset was identified using an integrated pipeline that combined LightGBM-based importance ranking, forward feature selection, and cross-validation. In the gradient bar plot, feature importance is represented via a Viridis color gradient (using standardized importance scores on a 0–1 scale). The selected features are highlighted with red axis labels for visual prominence. In the dual-axis line chart, candidate feature performance is measured by mean AUC values (represented by a solid red line with circular markers; 95% confidence intervals are shown as shaded areas), while black diamond markers indicate non-selected features.
